# Supplementary material for: A Putative Interaction between the Transmembrane Domains of Streptococcus pyogenes Sortase A and Its Endogenous Substrate M Protein Revealed by Molecular Dynamics Simulations
Source: J Phys Chem B. 2025 Dec 9;129(51):13117–27. doi: 10.1021/acs.jpcb.5c05800 (PMC12746448; doi:10.1021/acs.jpcb.5c05800)
Supplement: Supplementary file 1 [file jp5c05800_si_001.pdf]

Supporting Information for:

A Putative Interaction Between the Transmembrane Domains of *Streptococcus pyogenes* Sortase A and its Endogenous Substrate M Protein Revealed by Molecular Dynamics Simulations

Nathan G. Avery,[1] Elise F. Tahti,[1] Paul Clinton Spiegel,[1] John M. Antos,[1] James McCarty,[1] and Jeanine F. Amacher[1]

[1]Department of Chemistry, Western Washington University, Bellingham, Washington, 98225, USA

Table of Contents:

Supplemental Methods. S2

Table S1. Details of the molecular dynamics simulation size. S3

Figure S1. Prediction of transmembrane regions in spySrtA and M protein. S4

Figure S2. RMSF and RMSD values of triplicate molecular dynamics simulations for full-length spySrtA in a lipid bilayer. S5

Figure S3. AlphaFold3 modeling of spySrtA-M protein complexes. S6

Figure S4. RMSF and RMSD values of triplicate molecular dynamics simulations for full-length spySrtA with the LPSTG peptide in a lipid bilayer. S7

Figure S5. RMSF and RMSD values of triplicate molecular dynamics simulations for full-length spySrtA with M protein in a lipid bilayer, and averaged RMSF values. S8

Figure S6. Distribution of distances between the C208 thiol and P1 Thr carbonyl carbon for all simulations. S9

Figure S7. Control MD simulations of M protein alone or transmembrane domains alone. S10

Figure S8. Multiple sequence alignment of 28 *Streptococcus* SrtA sequences. S11-S12

Sequences used for AlphaFold2 modeling. S13

References S14

Supplemental Methods.

Molecular dynamics simulation parameters. Each system was solvated using TIP3P water and sodium and chloride ions to neutralize the system with an ionic strength of 0.15 M and equilibrated using GROMACS 2022.4 (**Table S1**).[1] A steepest decent energy minimization was performed until the maximum force on any atom is less than 1000 kJ/mol/nm. The temperature was first equilibrated at 300K with restraints on all heavy protein and lipid atoms using a Berendsen thermostat for 250 ps.[2] The pressure of the system was equilibrated in the NPT ensemble at 1 bar with decreasing restraints on all heavy protein and lipid atoms using a Berendsen semi-isotropic barostat for 1.75 ns total.[2] The temperature and pressure of the system was further equilibrated in the NPT ensemble with a Parrinello-Rahman semi-isotropic barostat[3] and Nose-Hoover thermostat[4,5] at 300K without restraints for 10 ns. Hydrogen atoms were restrained with the LINCS algorithm.[6] The equilibrated structures were used to run a single 500 ns simulation at 300K and 1 bar using GROMACS 2022.4.[1] Simulations were performed in triplicate, with separate equilibrations run for each. Atomic coordinates were saved every 100 ps.

Analyses of MD simulations. Contacts between atom pairs (spySrtA, DOPG, TOCL2, and/or substrate) were calculated with PLUMED 2.4.[7,8] A contact was considered formed if the distance between atoms was less than 4 Å. The contacts included nitrogen, oxygen, and carbon atoms for each residue in spySrtA near the membrane surface and nitrogen, oxygen, and carbon atoms for each lipid type in the membrane (DOPG and TOCL2). Contacts were measured between hydrophilic (nitrogen and oxygen) and hydrophobic (carbon) atoms in spySrtA and each lipid type, and/or between nitrogen, oxygen, and carbon atoms for each residue in spySrtA and substrate. Contacts were monitored over the 500 ns simulations. Distances between the transmembrane region of spySrtA and M protein substrate were measured with PLUMED from the center of geometry from residues (residues 13-37, spySrtA) and (387-409, M protein).

Root mean squared fluctuation (RMSF) per residue of backbone atoms, root mean squared deviation (RMSD) of backbone atoms, and solvent accessible surface area (SASA) per residue for side chain and backbone atoms were calculated using GROMACS analysis tools.[9] Potential energy between spySrtA and substrate or peptide was calculated using GROMACS.

Table S1. Details of the molecular dynamics simulation size.

System	Total number of atoms	Cubic box dimensions [nm]	Simulation time [ns], N=3 (T1,T2,T3)
SpySrtA (Apo)	89742	8.12781 x 8.12781 x 13.25244	500
SpySrtA-LPSTG	87925	8.04625 x 8.04625 x 13.20473	500
SpySrtA-M Protein	91827	8.18071 x 8.18071 x 13.41281	500
SpySrtA-TM-M Protein-TM	52411	8.17907 x 8.17907 x 8.50000	500
M Protein (Apo)	66125	8.12858 x 8.12858 x 10.77890	500

A

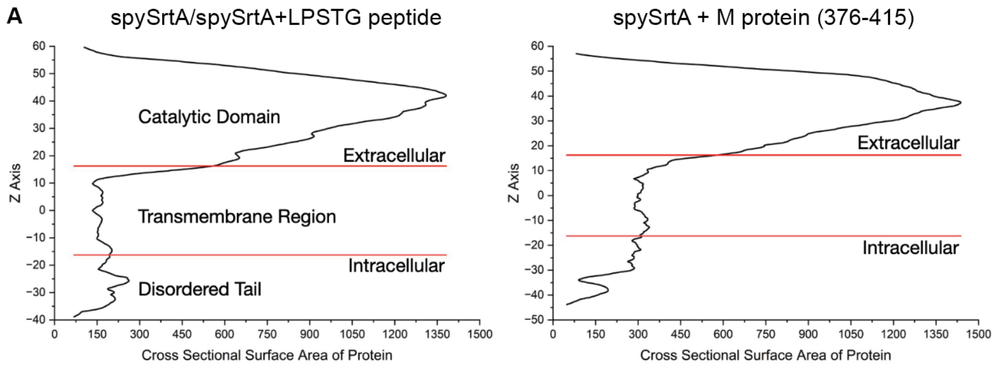

B

Construct	Depth/Hydrophobic Thickness (Å)	$\Delta G_{transfer}$ (kcal/mol)	Tilt Angle	Membrane Embedded Residues
spySrtA	30.2 ± 1.4	-28.7 kcal/mol	21.± 3.°	13-37,83
spySrtA and substrate	27.0 ± 1.9	-35.5 kcal/mol	18.± 1.°	14-34, 36-37, 47-48 (spySrtA)<br>384-402, 404 (M protein)

C

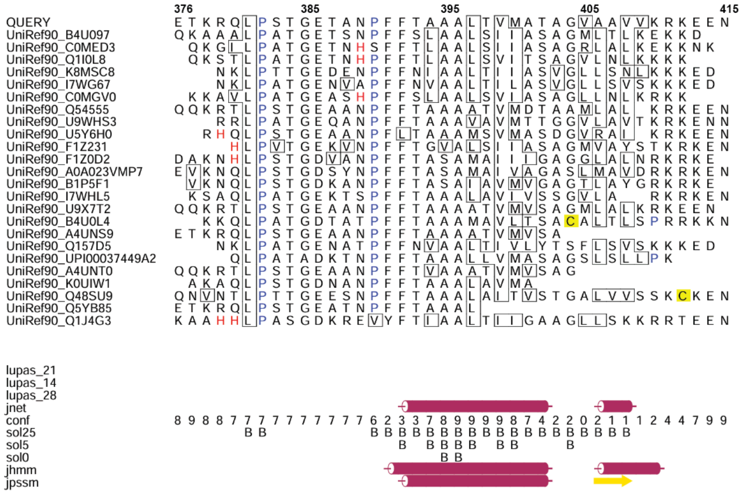

Figure S1. Prediction of transmembrane regions in spySrtA and M protein. PPM2.0 was used to predict the transmembrane domain boundaries for spySrtA in output AlphaFold2 models that either contained just full-length spySrtA or spySrtA plus an LPSTG peptide or extended M protein sequence, as labeled. (**A**) The cross sectional surface area of the protein is graphed as a function of the Z Axis, or distance from center of the lipid bilayer (defined as equal to 0). The globular extracellular domain is predicted to have a larger cross sectional surface area than either the transmembrane domain or disordered tail. **(B)** Details of membrane insertion of spySrtA with and without substrate. (**C**) Results of JPred4 secondary structure prediction for the M protein construct used in MD simulations, numbering is based on M protein (UniProt ID: M6A_STRP6). The C-terminus of the predicted transmembrane domain (^{205}VAAVV^{209}) is predicted by two output measures to contain α-helical character (bottom symbols), but it is not as conclusive as the region spanning ^{391}FFTAAALTVMAT^{402}.

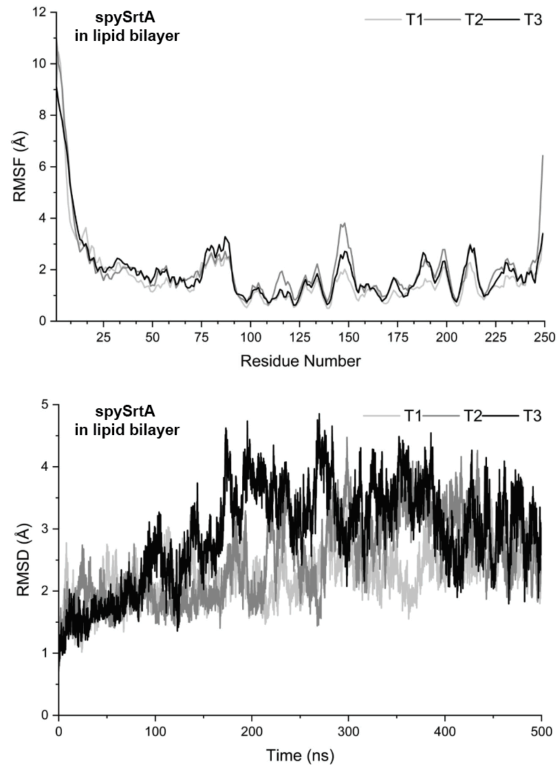

Figure S2. RMSF and RMSD values of triplicate molecular dynamics simulations for full-length spySrtA in a lipid bilayer. The root-mean-square-fluctuation (RMSF), or average displacement from their average position, for each residue (using the Cα atoms) in full-length spySrtA is shown for each simulation replicate: T1, T2, and T3 (**top**). This highlights regions of the protein with relatively higher degrees of flexibility. The room-mean-square-deviation (RMSD) for the averaged spySrtA protein over each 500 ns simulation is shown for each simulation replicate: T1, T2, and T3 (**bottom**).

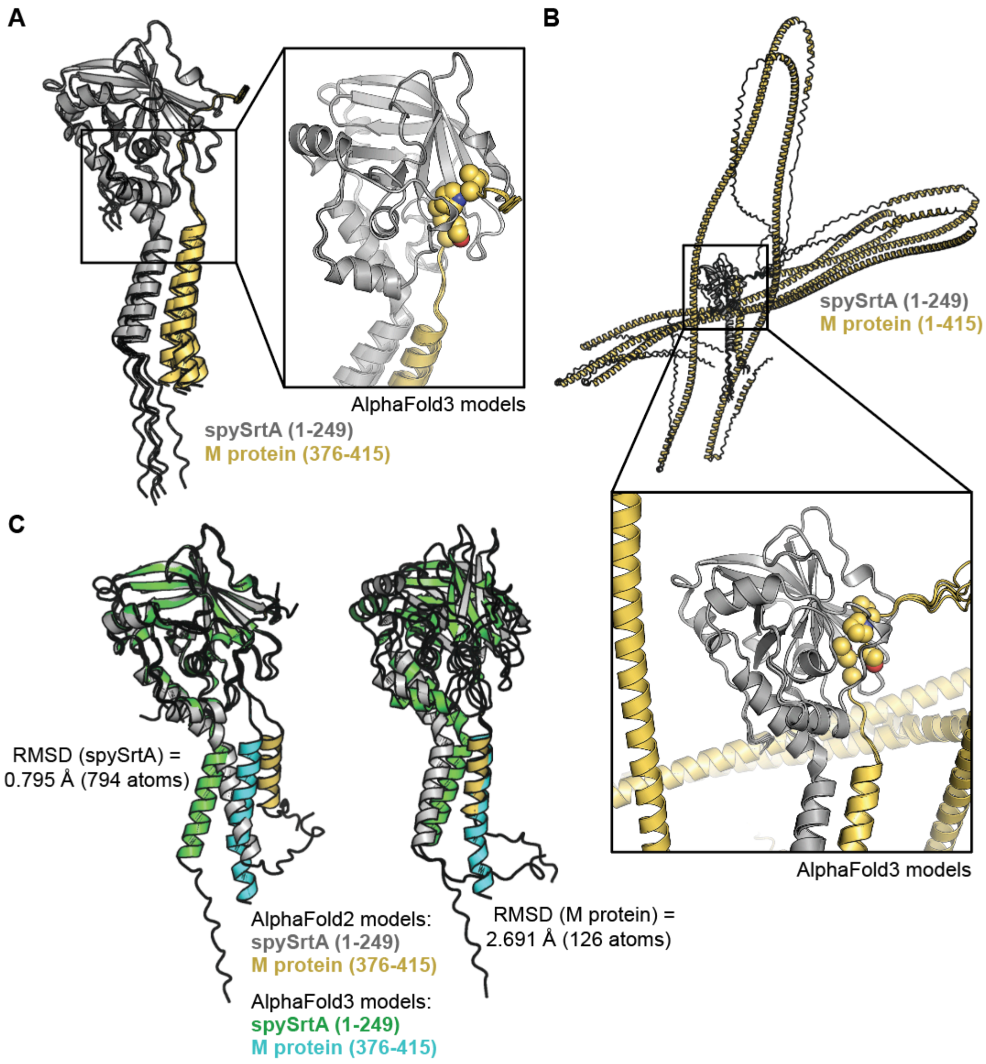

Figure S3. AlphaFold3 modeling of spySrtA-M protein complexes. The spySrtA protein is shown as gray cartoon. The M protein (either truncated or full-length) is shown in yellow cartoon representation. The LPSTG sortase recognition motif residues are in spheres and colored by atom (C=yellow, O=red, N=blue) in the zoomed in views to the right. (**A**) AlphaFold3 was used to model the spySrtA-M protein sequences used in MD simulations (ipTM=0.64, pTM=0.76). The spySrtA proteins from all 5 output models closely aligned, with RMSD values = 0.151 Å (1548 atoms), 0.114 Å (1492), 0.122 Å (1487), and 0.107 Å (1509), as compared to model_0, respectively. These models confirm that AlphaFold3 better predicts the full transmembrane domain helix of M protein than AlphaFold2. (**B**) AlphaFold3 was used to model the full-length spySrtA and full-length M protein sequences ((ipTM=0.59, pTM=0.36). The spySrtA proteins from all 5 output models closely aligned, with RMSD values = 0.132 Å (1550 atoms), 0.125 Å (1494), 0.135 Å (1470), and 0.116 Å (1500), as compared to model_0, respectively. A close-up view of the LPSTG sortase recognition binding site (right) confirms that only 2-3 amino acids N-terminal to the sortase recognition motif may interact with spySrtA, validating why the truncated M protein (residues 376-415) was used for MD simulations. (C) Alignment of the AlphaFold2 model (starting model of T1 replicate) as compared to the AlphaFold3 output_0 model. Proteins are in cartoon representation with the AlphaFold2 models in gray (spySrtA) and yellow (M protein), and AlphaFold3 models in green (spySrtA) and cyan (M protein). The spySrtA enzymes or M proteins were aligned by main chain atoms with RMSD = 0.795 Å (794) for spySrtA (left image) and RMSD = 2.691 Å (126) for M protein (right image).

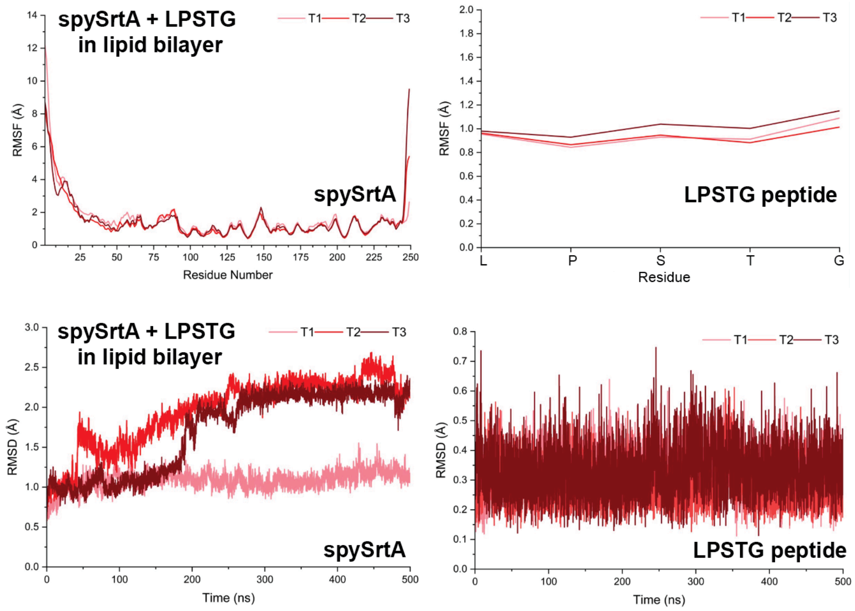

Figure S4. RMSF and RMSD values of triplicate molecular dynamics simulations for full-length spySrtA with the LPSTG peptide in a lipid bilayer. The root-mean-square-fluctuation (RMSF), or average displacement from their average position, for each residue (using the Cα atoms) in full-length spySrtA (**left**) or the LPSTG peptide (**right**) is shown for each simulation replicate: T1, T2, and T3 (**top**). The room-mean-square-deviation (RMSD) for the averaged spySrtA protein (**left**) or LPSTG peptide (**right**) over each 500 ns simulation is shown for each simulation replicate: T1, T2, and T3 (**bottom**).

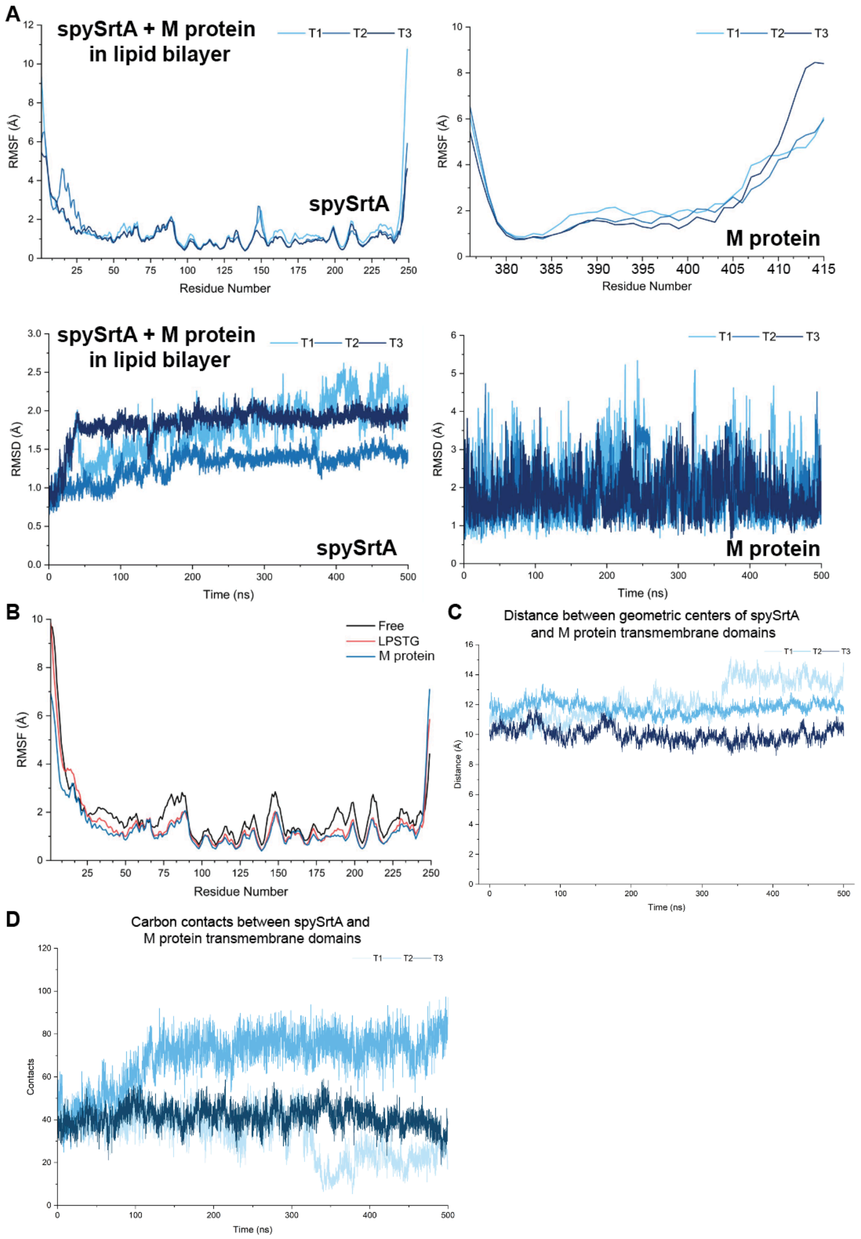

Figure S5. RMSF and RMSD values of triplicate molecular dynamics simulations for full-length spySrtA with M protein in a lipid bilayer, and averaged RMSF values. (**A**) The root-mean-square-fluctuation (RMSF), or average displacement from their average position, for each residue (using the Cα atoms) in full-length spySrtA (**left**) or M protein (**right**) is shown for each simulation replicate: T1, T2, and T3 (**top**). The room-mean-square-deviation (RMSD) for the averaged spySrtA protein (**left**) or M protein (**right**) over each 500 ns simulation is shown for each simulation replicate: T1, T2, and T3 (**bottom**). (**B**) Averaged RMSF values for spySrtA in the full-length spySrtA ("free"), spySrtA+LPSTG ("LPSTG"), and spySrtA+M protein ("M protein") triplicate simulations is shown. (**C**) Distance between the geometric centers of the spySrtA (residues 13-37) and M protein (residues 387-409) transmembrane domains for each replicate over the course of the simulations. (**D**) The number of carbon contacts (defined as <4 Å) over time for the spySrtA (residues 13-37) and M protein (residues 387-409) transmembrane domains for each replicate over the course of the simulations.

Distribution of distances between C208 thiol and P1 Thr carbonyl C

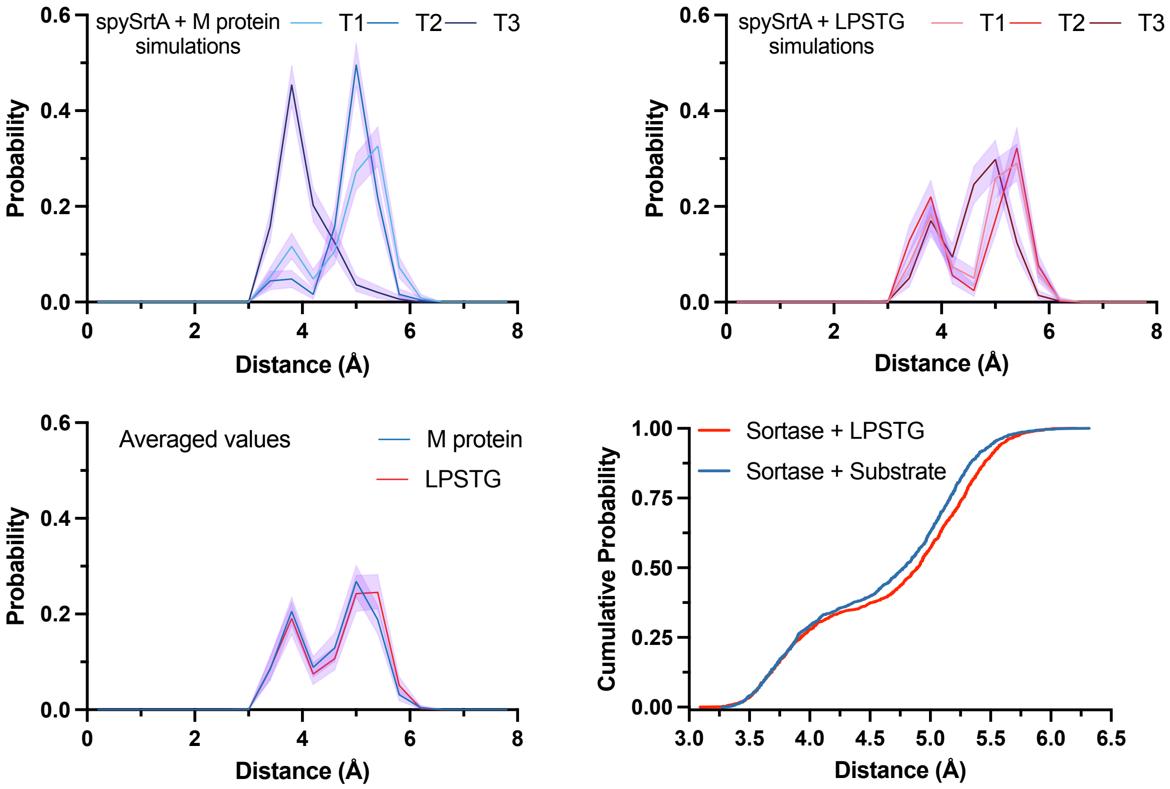

Figure S6. Distribution of distances between the C208 thiol and P1 Thr carbonyl carbon for all simulations. The distance for each state of a replicate simulation (T1, T2, and T3) between the C208 thiol and its site of nucleophilic attack, the P1 Thr carbonyl carbon, was graphed as a distribution and is shown for the spySrtA+LPSTG peptide simulations (**top left graph**) and spySrtA+M protein simulations (**top right graph**). Averaged values over triplicate simulations are shown in the **bottom left graph**, as labeled (spySrtA+LPSTG = red, spySrtA+M protein = blue). Bootstrapped 95% confidence intervals are indicated as purple shaded regions surrounding the probability values. Emperical cumulative distribution functions (ECDFs) from a two-sample Kolmogorov-Smirnov (KS) test comparing the distributions of distances between the C208 thiol and the P1 Thr carbonyl carbon from the pooled triplicate 500 ns simulations for spySrtA+LPSTG and spySrtA+M protein are shown in the **bottom right graph**. KS = 0.0873 and p-value = 2.1269×10^{-5}.

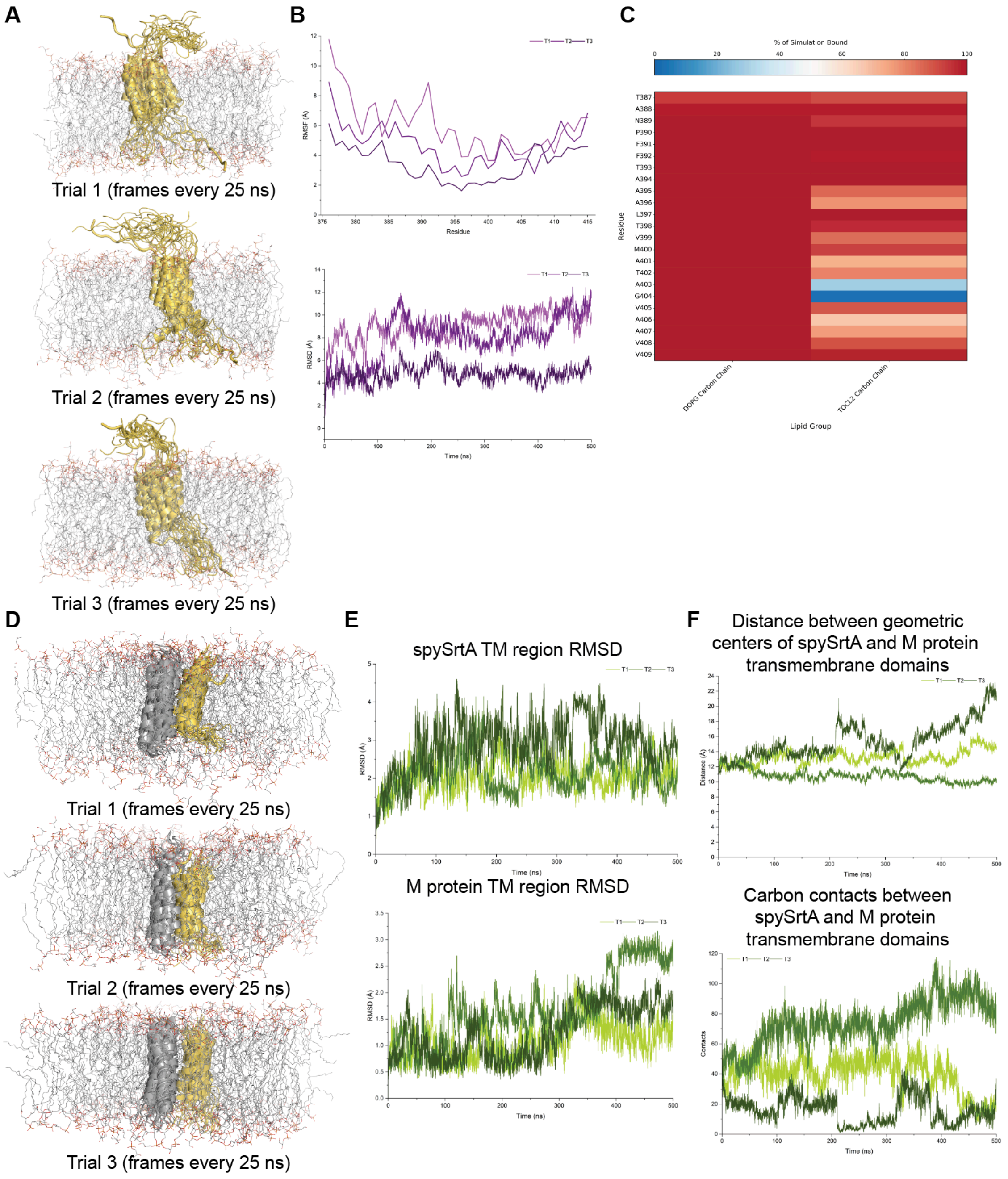

Figure S7. Control MD simulations of M protein alone or transmembrane domains alone. (**A, D**) Frames (every 25 nsec) of the triplicate simulations are shown for the M protein construct alone (**A**) and transmembrane domains control simulation (**D**). The presence of the spySrtA transmembrane domain appeared to stabilize the M protein transmembrane domain (**D**), as compared to it alone (**A**). Proteins are shown as cartoons (spySrtA = gray, M protein = yellow), and lipids are lines, and colored by heteroatom (C=gray, O=red). (**B, E**) Backbone RMSF and RMSD values (M protein, **B**) and RMSD values (**E**) reveal that these simulations are relatively stable throughout the 500 nsec. (**C**) Contact analysis shows that the M protein transmembrane residues do not significantly resposition in the membrane and maintain similar lipid contacts. (**F**) The distance between the geometric centers of the spySrtA and M protein transmembrane domains does not vary substantially in the 3 simulations, with more variation by the end of the T3 simulation (**top**). SpySrtA and M protein maintain carbon contacts between the transmembrane domains during the three replicate transmembrane domain simulations (**bottom**).

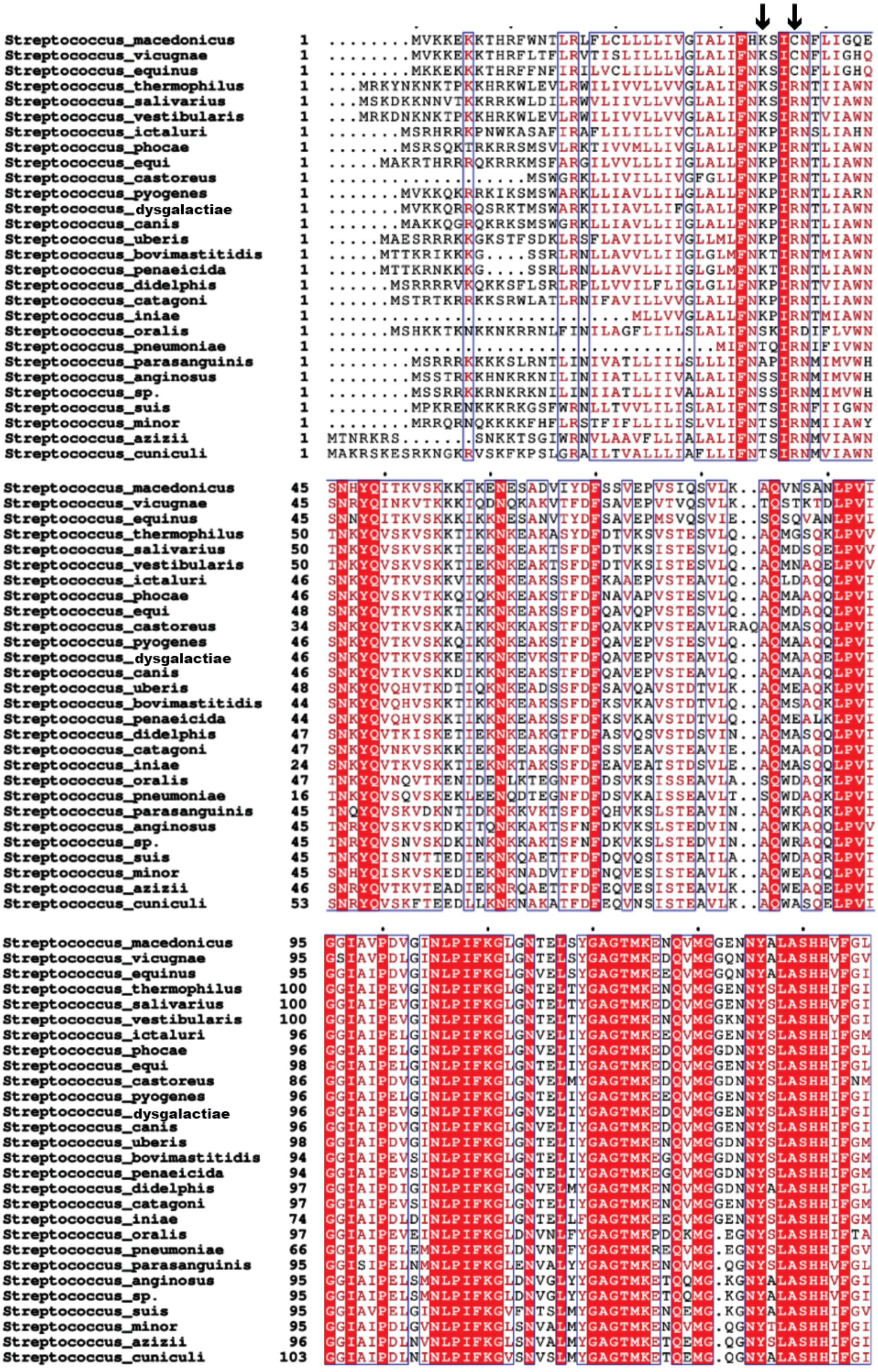

Streptococcus_macedonicus 1 ........MVKKEKKTHRFWNTLRLFLCLLLLIVGIALIFHKSICNFLIGQE
Streptococcus_vicugnae 1 ........MVKKEKKTHRFLTFLRVTISLILLLLGLALIFNKSICNFLIGHQ
Streptococcus_equinus 1 ........MKKEKKKTHRFFNFIRILVCLILLLVGLALIFNKSICNFLIGHQ
Streptococcus_thermophilus 1 ...MRKYNKNKTPKKHRKWLEVLRWILIVVLLVVGLALIFNKSIRNTIIAWN
Streptococcus_salivarius 1 ...MSKDKKNNVTKKRRKWLDILRWVLIIVLLVVGLALIFNKSIRNTVIAWN
Streptococcus_vestibularis 1 ...MRKDNKNKTPKKHRKWLEVLRWILIVVLLVVGLALIFNKSIRNTVIAWN
Streptococcus_ictaluri 1 .......MSRHRRKPNWKASAFIRAFLILILLIVCLALLFNKPIRNSLIAHN
Streptococcus_phocae 1 .......MSRSQKTRKRRSMSVLRKTIVVMLLIVGLALLFNKPIRNTLIAWN
Streptococcus_equi 1 .....MAKRTHRRRQKRRKMSFARGILVVLLLIIGLALLFNKPIRNTLIAWN
Streptococcus_castoreus 1 ...................MSWGRKLLIVILLIVGFGLLFNKPIRNTLIAWN
Streptococcus_pyogenes 1 .......MVKKQKRRKIKSMSWARKLLIAVLLILGLALLFNKPIRNTLIARN
Streptococcus_dysgalactiae 1 .......MVKKQRRQSRKTMSWARKILIAVLLIFGLALLFNKPIRNTLIAWN
Streptococcus_canis 1 .......MAKKQGRQKRKSMSWGRKLLIAVLLIIGLALLFNKPIRNTLIAWN
Streptococcus_uberis 1 .....MAESRRRKKGKSTFSDKLRSFLAVILLIVGLLMLFNKPIRNTLIAWN
Streptococcus_bovimastitidis 1 .....MTTKRIKKKG....SSRLRNLLAVVLLIIGLGLMFNKTIRNTMIAWN
Streptococcus_penaeicida 1 .....MTTKRNKKKG....SSRLRNLLAVVLLIIGLGLMFNKTIRNTLIAWN
Streptococcus_didelphis 1 ......MSRRRRVKQKKSFLSRLRPLLVVILFLIGLGLLFNKPIRNTVIAWN
Streptococcus_catagoni 1 ......MSTRTKRRKKSRWLATLRNIFAVILLVVGLALLFNKPIRNTLIAWN
Streptococcus_iniae 1 .............................MLLVVGLALLFNKPIRNTMIAWN
Streptococcus_oralis 1 ......MSHKKTKNKKNKRRNLFINILAGFLILLSLALIFNSKIRDIFLVWN
Streptococcus_pneumoniae 1 ...................................MIFNTQIRNIFIVWN
Streptococcus_parasanguinis 1 ........MSRRRKKKKSLRNTLINIVATLLIILSLLLIFNAPIRNMIMVWH
Streptococcus_anginosus 1 ........MSSTRKKHNKRKNILINIIATLLIIVALALIFNSSIRNMIMVWH
Streptococcus_sp. 1 ........MSSRRKKRNKRKNILINIIATLLIIVALALIFNSSIRNMIMVWH
Streptococcus_suis 1 ........MPKRENKKKRKGSFWRNLLTVVLILISLALIFNTSIRNFIIGWN
Streptococcus_minor 1 ........MRRQRNQKKKKFHFLRSTFIFLLILISLVLIFNTSIRNMIIAWY
Streptococcus_azizii 1 MTNRKRS.......SNKKTSGIWRNVLAAVFLLIALALIFNTSIRNMVIAWN
Streptococcus_cuniculi 1 MAKRSKESRKNGKRVSKFKPSLGRAILTVALLLIAFLLIFNTSIRNMVIAWN
Streptococcus_macedonicus 45 SNHYQITKVSKKKIKENESADVIYDFSSVEPVSIQSVLK..AQVNSANLPVI
Streptococcus_vicugnae 45 SNRYQINKVTKKKIQDNQKAKVTFDFSAVEPVTVQSVLK..TQSTKTDLPVI
Streptococcus_equinus 45 SNNYQITKVSKKKIKKNESANVTYDFSAVEPMSVQSVIE..SQSQVANLPVI
Streptococcus_thermophilus 50 TNKYQVSKVSKKTIKKNKEAKASYDFDTVKSVSTESVLQ..AQMGSQKLPVV
Streptococcus_salivarius 50 TNKYQVSKVSKKTIEKNKEAKTSFDFDTVKSISTESVLQ..AQMDSQELPVV
Streptococcus_vestibularis 50 TNKYQVSKVSKKTIEKNKEAKTSFDFDTVKSISTESVLQ..AQMNAQELPVV
Streptococcus_ictaluri 46 SNKYQVTKVSKKVIKKNKEAKSSFDFKAAEPVSTEAVLQ..AQLDAQQLPVI
Streptococcus_phocae 46 SNKYQVSKVTKKQIQKNKEAKSTFDFNAVAPVSTESVLQ..AQMAAQQLPVI
Streptococcus_equi 48 SNKYQVTKVSKKTIKKNKEAKSSFDFQAVQPVSTESVLQ..AQMDAQQLPVI
Streptococcus_castoreus 34 SNKYQVTKVSKKAIKKNKKAKSTFDFQAVKPVSTEAVLRAQAQMASQQLPVI
Streptococcus_pyogenes 46 SNKYQVTKVSKKQIKKNKEAKSTFDFQAVEPVSTESVLQ..AQMAAQQLPVI
Streptococcus_dysgalactiae 46 SNKYQVTKVSKKEIKKNKEVKSTFDFQAVEPVSTEAVLQ..AQMAAQELPVI
Streptococcus_canis 46 SNKYQVTKVSKKDIKKNKEAKSTFDFQAVEPVSTEAVLQ..AQMAAQQLPVI
Streptococcus_uberis 48 SNKYQVQHVTKDTIQKNKEADSSFDFSAVQAVSTDTVLK..AQMEAQKLPVI
Streptococcus_bovimastitidis 44 SNKYQVQHVSKKTIKKNKEAKSSFDFKSVKAVSTDTVLQ..AQMSAQKLPVI
Streptococcus_penaeicida 44 SNKYQVQHVSKKTIKKNKEAKSSFDFKSVKAVSTDTVLQ..AQMEALKLPVI
Streptococcus_didelphis 47 SNKYQVTKISKETIEKNKEAKGTFDFASVQSVSTDSVIN..AQMASQQLPVI
Streptococcus_catagoni 47 SNKYQVNKVSKKKIEKNKEAKGNFDFSSVEAVSTEAVIE..AQMAAQDLPVI
Streptococcus_iniae 24 SNKYQVTKVSKKTIEKNKTAKSSFDFEAVEATSTDSVLE..AQMASQQLPVI
Streptococcus_oralis 47 TNKYQVNQVTKENIDENLKTEGNFDFDSVKSISSEAVLA..SQWDAQKLPVI
Streptococcus_pneumoniae 16 TNKYQVSQVSKEKLEENQDTEGNFDFDSVKAISSEAVLT..SQWDAQKLPVI
Streptococcus_parasanguinis 45 TNQYQVSKVDKNTIDKNKKVKTSFDFQHVKSLSTEAVIN..AQWKAQKLPVI
Streptococcus_anginosus 45 TNRYQVSKVSKDKITQNKKAKTSFNFDKVKSLSTEDVIN..AQWKAQQLPVV
Streptococcus_sp. 45 TNRYQVSNVSKDKINKNKKAKTSFNFDKVKSLSTEDVIN..AQWRAQQLPVI
Streptococcus_suis 45 TNKYQISNVTTEDIEKNKQAETTFDFDQVQSISTEAILA..AQWDAQRLPVI
Streptococcus_minor 45 SNHYQISKVSKEDIEKNKNADVTFDFNQVESISTEAVLK..AQWEAQQLPVI
Streptococcus_azizii 46 SNRYQVTKVTEADIEKNRQAETTFDFEQVESISTEAVLK..AQWEAQELPVI
Streptococcus_cuniculi 53 SNRYQVSKFTEEDLLKNKNAKATFDFEQVNSISTEAVLK..AQWASQELPVI
Streptococcus_macedonicus 95 GGIAVPDVGINLPIFKGLGNTELSYGAGTMKENQVMGGENNYALASHHVFGL
Streptococcus_vicugnae 95 GSIAVPDLGINLPIFKGLGNTELSYGAGTMKEDQVMGGQNNYALASHHVFGV
Streptococcus_equinus 95 GGIAIPDVGINLPIFKGLGNVELSYGAGTMKEEQVMGGQNNYALASHHVFGI
Streptococcus_thermophilus 100 GGIAIPEVGINLPIFKGLGNTELTYGAGTMKENQVMGGENNYSLASHHIFGI
Streptococcus_salivarius 100 GGIAIPEVGINLPIFKGLGNTELTYGAGTMKEDQVMGGENNYSLASHHVFGI
Streptococcus_vestibularis 100 GGIAIPEVGINLPIFKGLGNTELTYGAGTMKENQVMGGKNNYSLASHHVFGI
Streptococcus_ictaluri 96 GGIAIPELGINLPIFKGLGNVELIYGAGTMKEEQVMGGENNYSLASHHIFGM
Streptococcus_phocae 96 GGIAIPELGINLPIFKGLGNVELIYGAGTMKEDQVMGGDNNYSLASHHIFGL
Streptococcus_equi 98 GGIAIPELGINLPIFKGLGNTELIYGAGTMKEDQVMGGENNYSLASHHIFGL
Streptococcus_castoreus 86 GGIAIPDVGINLPIFKGLGNVELMYGAGTMKEDQVMGGDNNYSLASHHIFNM
Streptococcus_pyogenes 96 GGIAIPELGINLPIFKGLGNTELIYGAGTMKEEQVMGGENNYSLASHHIFGI
Streptococcus_dysgalactiae 96 GGIAIPEVGINLPIFKGLGNVELIYGAGTMKEDQVMGGENNYSLASHHIFGI
Streptococcus_canis 96 GGIAIPDVGINLPIFKGLGNVELIYGAGTMKEDQVMGGENNYSLASHHIFGI
Streptococcus_uberis 98 GGIAIPDVGINLPIFKGLGNTELIYGAGTMKENQVMGGDNNYSLASHHIFGM
Streptococcus_bovimastitidis 94 GGIAIPEVSINLPIFKGLGNTELIYGAGTMKEGQVMGGDNNYSLASHHIFGM
Streptococcus_penaeicida 94 GGIAIPEVSINLPIFKGLGNTELIYGAGTMKEGQVMGGDNNYSLASHHIFGM
Streptococcus_didelphis 97 GGIAIPDIGINLPIFKGLGNVELMYGAGTMKENQVMGGDNNYALASHHIFGL
Streptococcus_catagoni 97 GGIAIPEVSINLPIFKGLGNTELIYGAGTMKENQVMGGENNYSLASHHIFGM
Streptococcus_iniae 74 GGIAIPDLDINLPIFKGLGNTELLFGAGTMKEEQVMGGENNYSLASHHIFGM
Streptococcus_oralis 97 GGIAIPEVEINLPIFKGLDNVNLFYGAGTMKPDQKMG.EGNYSLASHHIFTA
Streptococcus_pneumoniae 66 GGIAIPELEMNLPIFKGLDNVNLFYGAGTMKREQVMG.EGNYSLASHHIFGV
Streptococcus_parasanguinis 95 GGISIPELNMNLPIFKGLENVALYYGAGTMKENQVMG.QGNYSLASHHVFGL
Streptococcus_anginosus 95 GGIAIPELSMNLPIFKGLDNVGLYYGAGTMKETQQMG.KGNYALASHHVFGI
Streptococcus_sp. 95 GGIAIPELSMNLPIFKGLDNVGLYYGAGTMKETQQMG.KGNYALASHHVFGI
Streptococcus_suis 95 GGIAVPELGINLPIFKGVFNTSLMYGAGTMKENQEMG.KGNYALASHHIFGV
Streptococcus_minor 95 GGIAIPDLGVNLPIFKGLSNVALMYGAGTMKENQVMG.QGNYTLASHHIFGI
Streptococcus_azizii 96 GGIAIPDLNVNLPIFKGLSNVALMYGAGTMKETQEMG.QGNYSLASHHIFGI
Streptococcus_cuniculi 103 GGIAIPDLNINLPIFKGVSNVSLMYGAGTMKETQEMG.QGNYALASHHIFGI

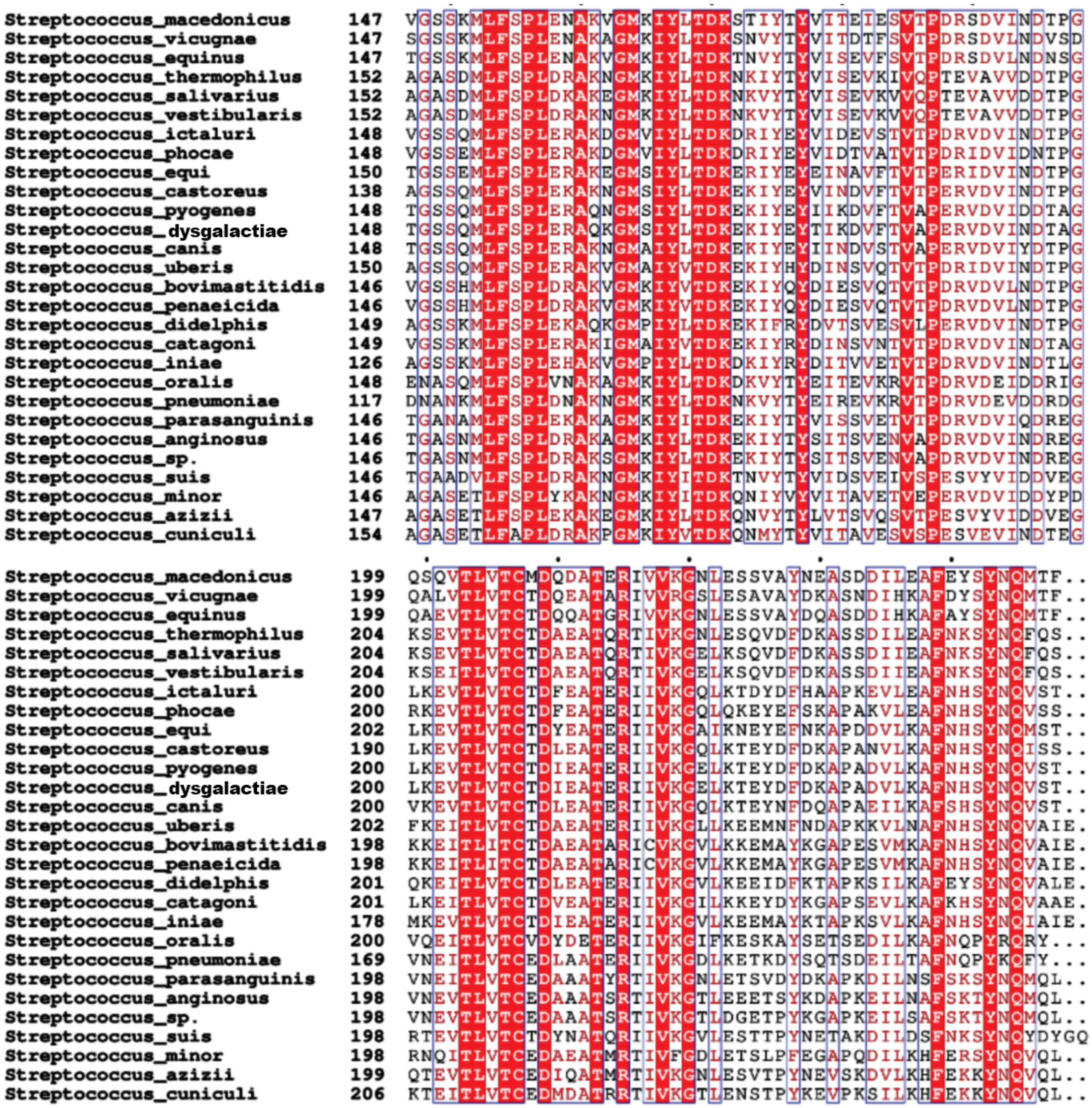

Figure S8. Multiple sequence alignment of 28 *Streptococcus* SrtA sequences. Multiple sequence alignment (MSA) was performed using Clustal Omega, as described in the Materials and Methods. The K35 and R38 spySrtA positions are indicated with black arrows.

Sequences used for AlphaFold2 modeling.

>spySrtA_Q99ZN4_STRP1_1-249
MVKKQKRRKIKSMSWARKLLIAVLLILGLALLFNKPIRNTLIARNSNKYQVTKVSKKQIKKNKEAKSTFDFQAVEPV
STESVLQAQMAAQQLPVIGGIAIPELGINLPIFKGLGNTELIYGAGTMKEEQVMGGENNYSLASHHIFGITGSSQML
FSPLERAQNGMSIYLTDKEKIYEYIIKDVFTVAPERVDVIDDTAGLKEVTLVTCTDIEATERIIVKGELKTEYDFDK
APADVLKAFNHSYNQVST

>M_protein_M6A_STRP6_376-415
ETKRQLPSTGETANPFFTAAALTVMATAGVAAVVKRKEEN

Sequences used for AlphaFold3 modeling also included full-length M protein.

>M_protein_M6A_STRP6_1-415
MAKNNTNRHYSLRKLKKGTASVAVALSVIGAGLVVNTNEVSARVFPRGTVENPDKARELL
NKYDVENSMLQANNDKLTTENKNLTDQNKELKAEENRLTTENKGLTKKLSEAEEEAANKE
QESKETIGTLKKILDETVKDKIAREQKSKQDIGALKQELAKKDEGNKVSEASRKGLRRDL
DASREAKKQVEKDLANLTAELDKVKEEKQISDASRKGLRRDLDASREAKKQVEKDLANLT
AELDKVKEEKQISDASRQGLRRDLDASREAKKQVEKALEEANSKLAALEKLNKELEESKK
LTEKEKAELQAKLEAEAKALKEQLAKQAEELAKLRAGKASDSQTPDAKPGNKVVPGKGQA
PQAGTKPNQNKAPMKETKRQLPSTGETANPFFTAAALTVMATAGVAAVVKRKEEN

References

(1) Berendsen, H. J. C.; van der Spoel, D.; van Drunen, R. GROMACS: A Message-Passing Parallel Molecular Dynamics Implementation. *Comput Phys Commun* **1995**, *91*, 43–56.

(2) Berendsen, H. J. C.; Postma, J. P. M.; van Gunsteren, W. F.; DiNola, A.; Haak, J. R. Molecular Dynamics with Coupling to an External Bath. *J. Chem. Phys.* **1984**, *81*, 3684-3690.

(3) Parrinello, M. Polymorphic Transitions in Single Crystals: A New Molecular Dynamics Method. *J. Appl. Phys.* **1981**, *52*, 7182-7190.

(4) Hoover, W. G. Canonical Dynamics: Equilibrium Phase-Space Distributions. *Phys. Rev. A, Gen. Phys.* **1985**, *31*, 1695–1697.

(5) Nosé, S. A Molecular Dynamics Method for Simulations in the Canonical Ensemble. *Mol. Phys.* **1984**, *52*, 255–268.

(6) Hess, B.; Bekker, H.; Berendsen, H. J. C.; Fraaije, J. G. E. M. LINCS: A Linear Constraint Solver for Molecular Simulations. *J. Comput. Chem.* **1997**, 1463-1472.

(7) PLUMED consortium. Promoting Transparency and Reproducibility in Enhanced Molecular Simulations. *Nat. Methods* **2019**, *16*, 670–673.

(8) Tribello, G. A.; Bonomi, M.; Branduardi, D.; Camilloni, C.; Bussi, G. PLUMED 2: New Feathers for an Old Bird. *Comput Phys Commun* **2014**, *185*, 604–613.

(9) Eisenhaber, F.; Lijnzaad, P.; Argos, P.; Sander, C.; Scharf, M. The Double Cubic Lattice Method: Efficient Approaches to Numerical Integration of Surface Area and Volume and to Dot Surface Contouring of Molecular Assemblies. *J. Comput. Chem.* **1995**, *16*, 273–284.
